# Agreement between arterial blood pressures measured non-invasively and invasively in anaesthetised sheep

**DOI:** 10.1186/s13028-025-00833-6

**Published:** 2025-12-08

**Authors:** Sanna Kaisa Sainmaa, Magdy Adam Hussein Adam, Daniela C. Casoni, Anna Vilhelmiina Huuskonen, Anna Valldeoriola Cardó, Marja Riitta Raekallio, Anna Kristina Mykkänen

**Affiliations:** 1 Korkeasaari Zoo, Mustikkamaanpolku 12, Helsinki, 00810 Finland; 2 Department of Equine and Small Animal Medicine, Faculty of Veterinary Medicine, University of Helsinki, Viikintie 47, Helsinki, 00014 Finland; 3 Present Address: Pharmacology Department, Faculty of Veterinary Medicine, Beni-Suef University, Beni-Suef, 62511 Egypt; 4 Experimental Surgery Facility, Experimental Animal Center (EAC), University of Bern, Bern, Switzerland; 5 Equine Clinical Studies, Diagnostic Imaging and Anaesthesia, UCD School of Veterinary Medicine, University College Dublin, Dublin, D04W6F6 Ireland

**Keywords:** Aneroid manometer, Doppler, Electronic pressure transducer, Oscillometric device

## Abstract

**Background:**

Both invasive and non-invasive blood pressure measuring methods are used in clinical and experimental work in veterinary medicine and several studies validating these methods are published. The aim of this study was to assess the level of agreement between non-invasive and invasive arterial blood pressure measurements within a wide range of blood pressures in sheep. Six adult Texel-cross female sheep were included. Anaesthesia was induced with IV propofol (4–8 mg/kg) and maintained with sevoflurane in 50% oxygen and air. Blood pressure measurements were simultaneously obtained using both invasive methods (electronic pressure transducer (EPT) connected to the auricular and carotid arteries, and aneroid manometer connected to the auricular artery) and non-invasive methods (the oscillometric device (OD) and Doppler). Carotid artery EPT was considered the ‘gold standard’ to which other methods were compared. The agreement between the two methods was evaluated with the Bland-Altman method, in conditions of normotension, hypertension (MAP > 160 mmHg, induced with phenylephrine), and hypotension (MAP < 50 mmHg, induced with acepromazine). The devices were evaluated using the American College of Veterinary Internal Medicine guidelines.

**Results:**

Carotid MAP values ranged from 37 to 192 mmHg. Mean bias and limits of agreement were − 2.8 mmHg and − 11–5.4 mmHg for the auricular EPT MAP; 5.1 mmHg and − 12.2–22.3 mmHg for the aneroid manometer MAP; − 2.7 mmHg and − 23.9–18.6 mmHg for the OD MAP; − 1.5 mmHg and − 20.1–23.2 mmHg for the Doppler SAP; respectively. Correlation coefficients for all methods were > 0.95.

**Conclusions:**

Doppler SAP and OD MAP demonstrated acceptable accuracy over a broad blood pressure range, supporting their utility for sheep as surrogate of invasive methods of measuring blood pressure. Auricular EPT was the best surrogate of central arterial pressure to be used in clinical conditions.

## Background

Invasive blood pressure (IBP) measurement methods that measure pressure directly from an artery are commonly regarded as the gold standard. They are more reliable compared to non-invasive blood pressure (NIBP) measurement techniques that are based on measuring changes in blood flow [1, 2]. For example, the auricular artery has commonly been used to measure IBP in both domestic [3] and nondomestic ruminants [4, 5]. However, catheterisation and securing the catheter in the artery may be both technically challenging and impractical in several clinical situations and requires aseptic preparation. Therefore, NIBP is often preferred in clinical veterinary medicine, particularly in field conditions or wildlife scenarios [6–8]. However, their accuracy across a wide range of blood pressures and validation in small ruminants remains largely understudied.

Invasive and non-invasive methods measure different physical properties. The invasive electronic pressure transducer (EPT) method, in which an arterial catheter is attached to a computed device through a fluid-filled tubing, responds to blood pressure changes which are then converted to changes in resistance and an electrical signal. The mean arterial pressure (MAP) is calculated from the area under the pressure curve, from which the systolic (SAP) and diastolic (DAP) arterial pressures are then calculated using an algorithm. Alternatively, MAP can be measured invasively with an aneroid manometer connected to an air- and fluid-filled tubing system and attached to an arterial catheter. The aneroid manometer system is pressurised to a level above the expected MAP, and the pressurised system is then allowed to equilibrate with the animal’s MAP [9]. The NIBP measurement methods, such as the oscillometric devices (OD) and the Doppler, measure changes in the blood pressure during external compression of an artery using an inflatable cuff. The OD measures MAP by detecting the maximum oscillations of pressure in the cuff, and estimates SAP and DAP with a proprietary algorithm [10]. The Doppler estimates SAP by measuring the threshold of regaining blood flow when compression of the artery is gradually lowered [9].

Doppler method to measure NIBP has limit of accuracy and precision in many species, such as dogs [11], cats [12, 13], rabbits [14] and goats [15]. However, it is considered to be an acceptable monitoring method for hypotensive small dogs and rabbits in general anesthesia [16, 17]. Various ODs have been used in nondomestic goats in the field [7, 18]. The OD has provided MAP measurements that were acceptable by the American College of Veterinary Internal Medicine (ACVIM) standards, albeit somewhat underestimated [19], in anaesthetised sheep [20] and adult goats [15]. However, in another study with a small sample size, the ACVIM recommendations [19] were not met in sheep [21]. According to the authors’ knowledge, there are two published validation studies of the invasive aneroid manometer method: one using a ‘pressure veil’ device in conscious cattle [22], and another performed on anaesthetised horses [23], but the method has not been validated in small ruminants. Additionally, the accuracy and precision of IBP measurement from the auricular artery have not been compared against measurements in a central artery in sheep. Furthermore, the previous studies comparing various methods to measure blood pressure in small ruminants focused on a relatively narrow range of blood pressures and therefore their applicability in situations with marked hypo- or hypertension is limited.

This study aims to compare various IBP and NIBP methods (EPT attached to the auricular artery, aneroid manometer attached to the same auricular artery, OD, and Doppler) for measuring blood pressures in normo-, hypo- and hypertensive animals during general anaesthesia, using carotid EPT as a reference. In this study, domestic sheep were used as a model for small ruminants for practical reasons.

## Methods

### Animals

Six Texel-cross sheep, 1.5–3.5 years of age, and a mean ± SD body weight of 55 ± 4 kg, were used in this study. The right carotid artery of each animal was previously surgically elevated into a subcutaneous position to facilitate catheterisation [24]. The animals were deemed healthy based on physical examination, haematology, and blood biochemistry results. Feed was withheld for 24 h prior to anaesthesia but the animals had free access to drinking water. The study was approved by the Finnish Animal Experiment Board (ESAVI/9394/04.10.07/2015).

### Study design

Prospective experimental study.

### Anaesthetic protocol

An intravenous 18 G indwelling catheter (Vygon, UK) was placed in the cephalic vein of the sheep while awake. After pre-oxygenation through an untight mask general anaesthesia was induced with intravenous administration of propofol (Vetofol, 10 mg/mL, Vetmedic, Finland) to effect (total dose: 4–8 mg/kg). Tracheal intubation was carried out with a silicone endotracheal tube (9–11 mm internal diameter, MILA International, KY, USA) under laryngeal visualisation and with the help of a bougie in sternal recumbency. After tracheal intubation, sheep were lifted onto a table and positioned in sternal recumbency.

General anaesthesia was maintained with sevoflurane (SevoFlo, Abbott Laboratories, IL, USA) in 50% mixture of oxygen and air, and the end-tidal sevoflurane concentration was stabilised at 3.0%. Intermittent positive pressure ventilation was established immediately after tracheal intubation using volume-controlled pressure- limited mode (Perseus A 500, Dräger, Germany). Positive end-expiratory pressure was set at 5 cmH_2_O and maintained constant throughout the anaesthesia. Maximum inspiratory pressure was set at 25 cmH_2_O and the inspiratory-expiratory ratio at 1:2. Tidal volume of 10 ml/kg was set at the beginning of anaesthesia and altered as needed, together with the respiratory rate (10–13 breaths per minute), aiming at normocapnia (EtCO_2_ 35–40 mmHg). All parameters were recorded every five minutes.

### Instrumentation

#### Electronic pressure transducer

A catheter was aseptically placed in the right carotid artery (20 G; B. Braun Melsungen, Germany) and left or right auricular artery on the side without an ear tag (22 G; B. Braun Melsungen). Both catheters were then connected by a fluid-filled non-compliant tubing to an electronic pressure transducer (Gabarith PMSET; Becton Dickinson, UT, USA), placed at the level of the right atrium and zeroed to atmospheric pressure. MAP, SAP, and DAP measurements were displayed on a compact critical care monitor (S/5 Datex-Ohmeda, GE Healthcare, Finland).

#### Aneroid manometer

Aneroid manometer attached to the same auricular artery as the auricular EPT was used to measure invasive MAP. The manometer was calibrated against a mercury manometer. The aneroid manometer was connected to a ‘pressure veil’ device, which in turn was attached to a sterile air-filled non-compliant tubing. The non-compliant extension tubing was connected to a three-way tap; then to another air-filled extension tubing; another three-way tap; and finally, to a third extension tubing primed with sterile saline which was attached to the intra-arterial catheter via a third three-way tap. To maximally prevent contamination of the manometer with saline and blood, we used a commercially made device (Pressure Veil, no longer manufactured) that resembles a rubber condom inside a rigid plastic syringe case, although the precaution might be redundant if a sufficient length of air-filled tubing is present between the saline-primed tubing and the manometer. An empty syringe was connected to the first three-way tap close to the manometer, in order to allow the removal of excess fluid from the system. Another, sterile saline-filled syringe was attached to the second three-way tap, which was open to the manometer and closed to the animal. Just prior to taking the measurement, sterile saline was injected towards the manometer, until the system was pressurised so that the manometer read a higher pressure than the expected MAP. The air-fluid interface was then positioned at the level of the right atrium, the third three-way tap opened to the animal, and the arterial pressure was allowed to communicate with the manometer via the fluid and air columns. After a short equilibration period the manometer pressure equalised with the MAP and was recorded.

#### Oscillometric device

An OD (PetMAP graphic, Ramsey Medical, FL, USA) was used with the cuff placed over the left radial artery. The cuff was at the level of the heart when the animal was lying in sternal recumbency. The width of the selected cuff was approximately 40% of the limb’s circumference. The device setting for dog front limb was selected.

#### Doppler

An ultrasonic Doppler flow detector (Doppler Medical Electronics 811-B, Parks Medical Electronics, OR, USA) was used to measure SAP. The flow probe was placed parallel to the direction of the flow in the digital plantar common artery, while the inflatable cuff was placed around the right metatarsus. The width of the cuff was 30–40% of the circumference of the metatarsus. The cuff was inflated intermittently to occlude the flow of the artery and thereafter progressively deflated until the arterial flow was acoustically detected again. The pressure measured on the manometer when the arterial flow returned was recorded as the SAP.

### Measurements and blood pressure manipulations

After instrumentation, baseline blood pressures were measured with all methods. To create variation in the values, the blood pressure was increased stepwise with phenylephrine hydrochloride (0.06, 0.12 and 0.24 mg/kg/h IV, Fenylefrin Unimedic, Unimedic Pharma, Sweden) until hypertension was reached (final target MAP > 160 mmHg). Thereafter blood pressure was decreased stepwise with acepromazine maleate (0.3–0.5 mg/kg IV, 3–4 doses repeatedly, Plegicil vet, Bela-Pharm, Vechta, Germany) until hypotension was achieved (final target MAP < 50 mmHg). At baseline and after each dose, the measurements were repeated five times with one-minute intervals with carotid EPT, auricular EPT, OD, and Doppler, simultaneously. The aneroid manometer was used for 1–3 times in between the auricular EPT measurements at each level due to it being a more time-consuming method. The animals were euthanised at the end of the anaesthesia with IV injection of pentobarbital (Euthasol Vet, Le Vet, Netherlands).

### Statistical methods

The level of agreement between methods was investigated using the Bland and Altman method for repeated measurements [25]. The blood pressure data from all measurement methods were compared to the carotid EPT data. All observations were considered as individual data points.

Bland-Altman plots presenting the difference between measurements taken with different methods against the mean of the measurements, and scatter plots with regression lines were created (https://www.medcalc.org/). Additionally, Pearson’s correlation coefficients, standard deviation (SD), mean bias, limits of agreement (LoA) and 95% confidence intervals (CI) for mean bias and exact parametric CI for LoAs were calculated for all comparisons with a formula provided by Carkeet [26].

Because Bland Altman method does not define whether the agreement interval is sufficient for clinical purposes, we used ACVIM recommendations [19] as guidelines. According to the ACVIM recommendation [19], 50% of all measurements for SAP and DAP treated separately should lie within 10 mmHg and 80% within 20 mmHg of the reference method. These proportions were also calculated for the MAP in addition to DAP and SAP.

## Results

The invasive MAP measured via the carotid EPT ranged from 37 to 192 mmHg during the monitoring period. In one sheep, the OD failed to measure blood pressure when carotid SAP was 54 mmHg or lower and DAP 38 mmHg or lower, but the device succeeded in another sheep with equally low pressures.

**Fig. 1:**
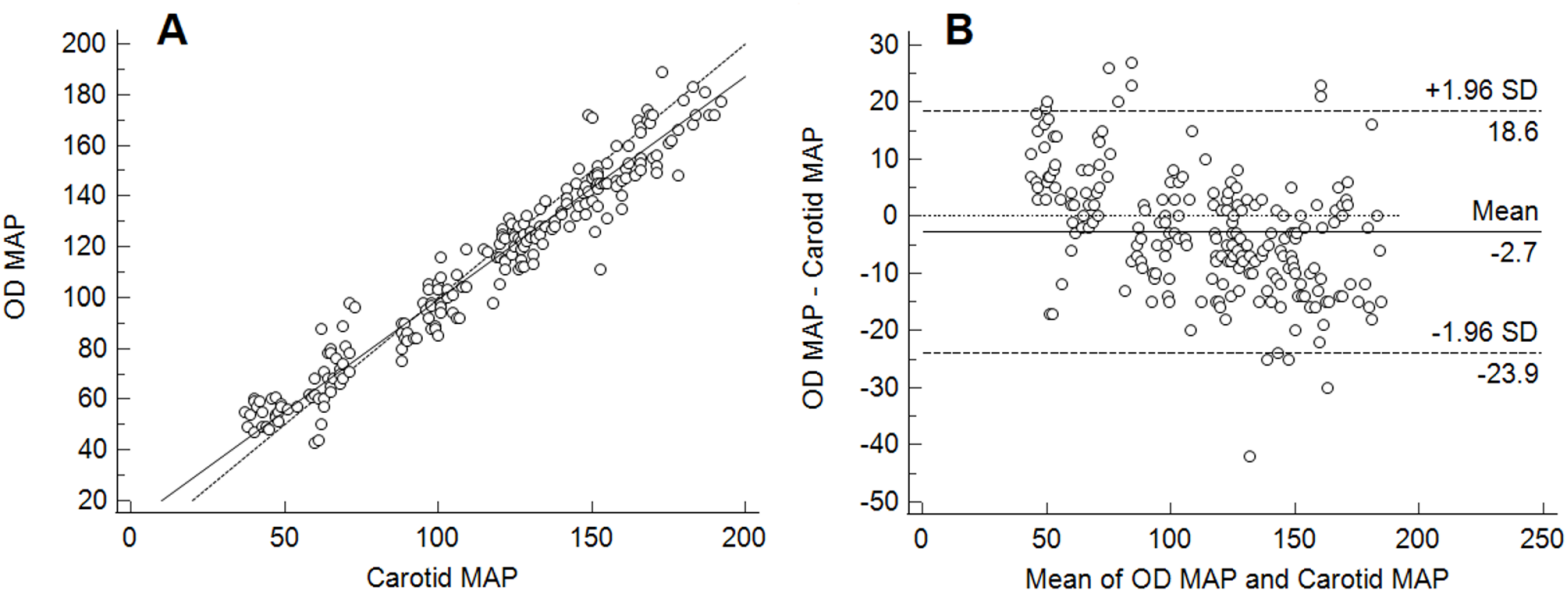
Comparison of mean arterial pressure measured with oscillometric device and electronic pressure device (carotid artery). **a** Scattered plots with regression line (solid line) and line of equality (dashed line). OD = oscillometric device. MAP = mean arterial pressure. **b** Bland-Altman plots describing agreement between two methods. Upper and lower limits of agreement calculated by using the mean bias and the standard deviation (SD) of the differences between two measurements (*n* = 217)

**Fig. 2:**
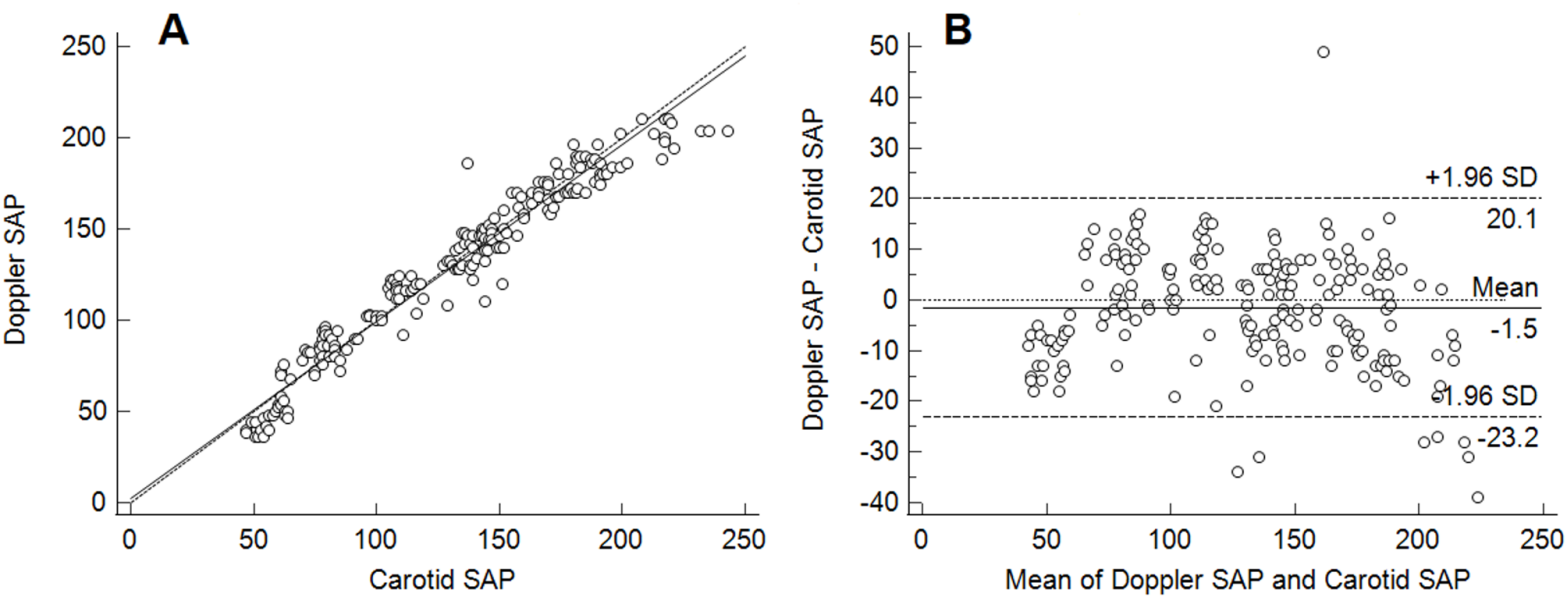
Comparison of mean arterial pressure measured with Doppler and electronic pressure device (carotid artery). **a** Scattered plots with regression line (solid line) and line of equality (dashed line). MAP = mean arterial pressure. **b** Bland-Altman plots describing agreement between two methods. Upper and lower limits of agreement calculated by using the mean bias and the standard deviation (SD) of the differences between two measurements (*n* = 218)

**Fig. 3:**
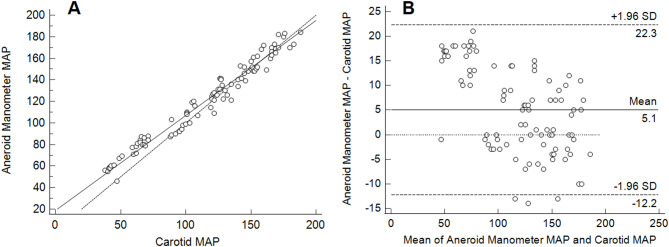
Comparison of mean arterial pressure measured with aneroid manometer and electronic pressure device (carotid artery). **a** Scattered plots with regression line (solid line) and line of equality (dashed line). MAP = mean arterial pressure. **b** Bland-Altman plots describing agreement between two methods. Upper and lower limits of agreement calculated by using the mean bias and the standard deviation (SD) of the differences between two measurements (*n* = 94)

**Fig. 4:**
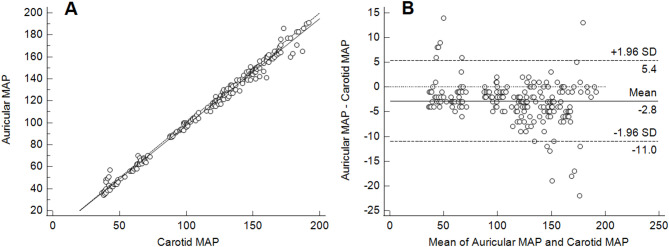
Comparison of mean arterial pressure measured with electronic pressure device from auricular and carotid artery. **a** Scattered plots with regression line (solid line) and line of equality (dashed line). MAP = mean arterial pressure. **b** Bland-Altman plots describing agreement between two methods. Upper and lower limits of agreement calculated by using the mean bias and the standard deviation (SD) of the differences between two measurements (*n* = 220)

The comparison of aneroid manometer, OD MAP, and Doppler with carotid EPT data are introduced as scatter plots with regression lines and Bland-Altman plots with mean, upper and lower LoAs (Figs. 1, 2, 3 and 4). Table 1 presents estimated mean bias (SD, CI), LoAs (CI), for all measurements. Among all methods, auricular EPT demonstrated the most accurate results compared to the carotid EPT. All invasive blood pressures measured with the auricular EPT, as well as the aneroid manometer MAP, the OD MAP, and the Doppler SAP met the ACVIM recommendations [19].

**Table 1:** Summary of results

Variable	*N*	Mean bias (SD) mmHg	CI for mean bias mmHg	Lower LoA (CI) mmHg	Upper LoA (CI) mmHg	*r*	Difference ≤ 10%	Difference ≤ 20%
OD DAP	217	− 13.3 (11.3)	− 14.8 to − 11.9	− 35.6 (− 37.9 to −33.7)	8.9 (7−11.3)	0.956	32.7	73.3
OD MAP	217	− 2.7 (10.8)	− 4.1 to − 1.2	− 23.9 (− 26.2 to −22.1)	18.6 (16.8–20.8)	0.971	64.5	95.4
OD SAP	217	15.9 (13.2)	14.2 to 17.7	− 10.0 (− 12.8 to − 7.8)	41.9 (39.7–44.6)	0.963	27.6	59.0
Auricular EPT DAP	220	− 3.6 (3.3)	− 4.1 to − 3.2	− 10.2 (− 10.9 to − 9.6)	2.9 (2.4–3.6)	0.996	97.3	100.0
Auricular EPT MAP	220	− 2.8 (4.2)	− 3.4 to − 2.3	− 11.0 (− 11.9 to − 10.3)	5.4 (4.7–6.2)	0.996	94.5	99.5
Auricular EPT SAP	220	− 0.8 (6.1)	− 1.6 to 0	− 12.8 (− 14 to − 11.8)	11.1 (10.1–12.4)	0.995	94.1	98.6
Doppler SAP	218	− 1.5 (11.0)	− 3 to 0	− 20.1 (− 25.5 to − 21.3)	23.2 (18.3–22.4)	0.974	68.8	96.3
Aneroid manometer MAP	94	5.1 (8.8)	3.3 to 6.9	− 12.2 (− 15.2 to − 10.1)	22.3 (20.2– 25.3)	0.983	64.2	99.0
ACVIM recommendations		< ± 10 ( < ± 15)				> 0.9	≤ 50	≤ 90

The mean bias and its standard deviation (SD) and confidence interval (CI), Bland-Altman limits of agreement (LoA) with confidence intervals and Pearson correlation coefficients (r) compared to the corresponding value measured invasively from the carotid artery, and percentage value of samples that were within 10 mmHg and 20 mmHg of the reference method. OD = oscillometric device, EPT = electronic pressure transducer

## Discussion

In this study, a wide range of blood pressures was induced to evaluate the accuracy of various measurement techniques in normo-, hypo-, and hypertensive sheep. As expected, the invasive blood pressure measured with the auricular EPT yielded the most accurate results compared to the invasive carotid artery pressures that were used as the reference. More than 90% of all measurements taken with the auricular EPT lied within 10 mmHg of the reference. In contrast, less than 70% of measurements from other methods fell within the 10 mmHg margin.

In our study, the adapted ACVIM criteria [19] were met for MAP by the aneroid manometer method. This result conforms with findings from a study performed on conscious cattle, where almost perfect agreement was observed between the aneroid manometer and an EPT when both were connected to the same auricular artery [22]. Nevertheless, our study found that the aneroid manometer MAP was overestimated in hypotensive sheep, in contrast to a previous study performed on anaesthetised horses, which reported that the MAP value given by the aneroid manometer was slightly underestimated compared to the control method [23]. This discrepancy may be due to differences in reference arteries. In the horse study an aneroid manometer was connected to the dorsal metatarsal artery, and it used an EPT attached to the facial artery as the control [23]. The MAP decreases when the blood flows from central arteries to peripheral arteries [27–29], and an earlier study in horses demonstrated that blood pressure values measured in different peripheral arteries cannot be used interchangeably [28]. Furthermore, fewer measurements were performed with the aneroid manometer compared to the other methods, potentially limiting statistical power when comparing the aneroid manometer with the carotid EPT. The time-consuming nature and the complex setup of the aneroid manometer method also introduced some delays in the timing of measurements. Although both IBP methods had a good overall agreement with the reference method, the overestimation of blood pressures in hypotensive sheep by the aneroid manometer may be marked.

In our study, the OD equipment appeared to be easy to use. The manufacturer recommends the OD for cats and dogs when the MAP is in the range of 30–260 mmHg. All blood pressures measured in our study were within this recommended range. Although the OD gave acceptable MAP values, the DAP and SAP values failed to satisfy the ACVIM recommendations [19]. Since the device measures MAP and estimates SAP and DAP [10] with an algorithm designed for dogs and cats, it is likely that this algorithm was not accurate for sheep. In earlier studies with non-invasive measurement techniques in dogs, errors have occurred in both directions [30, 31]. Although in our study, the CI for the mean bias suggested that OD systematically underestimated MAP, a minor trend to overestimate low MAP and underestimate high MAP was seen in the plots. MAP is a physiologically important blood pressure parameter representing the mean driving pressure for organ perfusion [9]. Therefore, the OD can be a useful method to measure MAP in small ruminants in a clinical setting. However, clinical decisions should not be made based on a single measurement of MAP due to the wide LoAs and tendency for systematic error in both low and high blood pressures.

Similar to the present study, some previous studies suggested the OD to be useful when considering its limitations [15, 20, 21] while others do not recommend its use in sheep and goats [32, 33]. Comparing our results to earlier ones that used the OD techniques in small ruminants is difficult because research setups vary considerably between studies. Some earlier studies used different equipment [15, 20, 32] and auricular [20, 21, 32] or femoral arteries [15] as a reference. The number of animals and measurements per animal also varied markedly between the studies. Furthermore, the range of blood pressures in the previous studies was narrower compared to our study, which may have affected the interpretation of the results. Since each OD device type can have different algorithms, the accuracy of different OD devices should be individually determined by comparison to a reference standard. Hence, our results can be applied only to the device type used in this study.

The Doppler measures only SAP values, which in our study satisfied the ACVIM recommendations [19]. The visual inspection of the Bland-Altman plots revealed a symmetrical scatter of results in both directions resulting relatively equal lower and upper LoAs in both directions. Although the mean bias was low and the CI of the mean bias was narrow, this cannot be interpreted as good precision in this case. Therefore, clinical decisions should be based on multiple measurements making Doppler acceptable for evaluating blood pressure trends during sheep anaesthesia. This finding was in contrast with an earlier study reporting an even wider LoA and not recommending it as an alternative for NIBP measurements in goats [15].

In our study, all methods had a correlation coefficient of >0.95 compared with Carotid EPT. This high correlation is likely influenced by the broad range of blood pressures achieved through pharmacological manipulation. However, correlation analysis has been criticised as an inappropriate method for evaluating agreement between two techniques measuring the same physiological parameter, as it assesses association rather than agreement [34]. Hence other values in this study should be emphasized over correlation when interpreting results.

A limitation of this study is the temporal mismatch between measurements taken with different devices. The sampling rate and the time required for a single measurement were slower with the aneroid manometer, the OD, and the Doppler, when compared with the auricular or carotid EPT. Therefore, those measurements were most likely not taken simultaneously with the reference measurement but typically the difference was less than a minute. This delay increased the variation in the results, because the blood pressure was manipulated over time in this study design. Furthermore, only six animals were recruited in our study. The ACVIM recommends at least eight animals to be used when comparing non-invasive measurement techniques to invasive ones [19]. However, in this study, we manipulated the blood pressure to reach a wide range of pressures in each animal and achieved a large total number of measurements, increasing the study power. Additionally, the ACVIM recommendations are created for validating NIBP devices and criteria for invasive methods such as aneroid manometer and auricular EPT can be too loose. Furthermore, we used ACVIM recommendations created for SAP and DAP values in a lack of other applicable criteria in veterinary medicine.

## Conclusions

In this study, the invasive auricular EPT pressures, the invasive aneroid manometer MAP, the OD MAP, and the Doppler SAP met the ACVIM recommendations. In addition to the invasive methods, the OD MAP and the Doppler SAP can be used in a wide range of blood pressures in sheep. Future studies should address limitations and standardise methodologies to further improve non-invasive measurement accuracy in sheep and other small ruminants.

## Data Availability

The datasets used and/or analysed during the current study are available from the corresponding author on reasonable request.

## References

[1] Valerio F, Mariscoli M, Petrizzi L. Comparative evaluation of the accuracy of oscillometric and direct methods for arterial blood pressure monitoring during anaesthesia in dogs. Vet Res Comm. 2006;30:321–3.

[2] Shih A, Robertson S, Vigani A, Pablo L, Bandt C. Evaluation of an indirect oscillometric blood pressure monitor in normotensive and hypotensive anesthetized dogs. J Vet Emerg Crit Care. 2010;20:313–8. 10.1111/j.1476-4431.2010.00536.x 20636984

[3] Fernández LG, Niimura del Barrio MC, Loughran C. Use of adrenaline continuous infusion to treat hypotension during general anaesthesia in a cow and a calf. Ir Vet J. 2020;73:13. 32637073 10.1186/s13620-020-00164-0 PMC7333263

[4] Einwaller J, Painer J, Raekallio M, Gasch K, Restitutti F, Auer U, et al. Cardiovascular effects of intravenous Vatinoxan (MK-467) in medetomidine-tiletamine-zolazepam anaesthetised red deer (*Cervus elaphus*). Vet Anaesth Analg. 2020;47:518–27. 32507716 10.1016/j.vaa.2019.10.011

[5] Rauch H, Pohlin F, Einwaller J, Habe M, Painer J, Stalder G. Comparison of the cardiovascular effects of two Medetomidine doses combined with tiletamine-zolazepam for the immobilization of red deer Hinds (*Cervus elaphus*). J Wildl Dis. 2022;58:188–93. 34724568 10.7589/JWD-D-20-00229

[6] Siegal-Willott J, Citino SB, Wade S, Elder L, Hayek LC, Lance WR. Butorphanol, azaperone, and Medetomidine anesthesia in free-ranging white-tailed deer (*Odocoileus virginianus*) using radiotransmitter darts. J Zoo Wildl Med. 2015;46:291–97. 19395756 10.7589/0090-3558-45.2.468

[7] Sainmaa S, Mykkänen A, Adam M, Jantunen N, Vainio O, Raekallio M. Intravenous Vatinoxan in Markhors (*Capra Falconeri heptneri*) immobilized with intramuscular Medetomidine and ketamine — a preliminary dose screening study. J Zoo Wildl Med. 2019;50:159–66. 31120674 10.1638/2018-0030

[8] Meuffels J, Lueders I, Bertschinger H. Cardiopulmonary parameters and arterial blood gases during etorphine-medetomidine-midazolam immobilization in free-ranging black rhinoceroses (*Diceros bicornis*) undergoing electro-ejaculation—A preliminary study. Front Vet Sci. 2021. 10.3389/fvets.2021.740614. 34926635 10.3389/fvets.2021.740614 PMC8674947

[9] Odette O. Blood pressure monitoring. In: Lamont LA, Grimm KA, Robertson S, et al. editors. Veterinary anaesthesia and analgesia: the sixth edition of Lumb and Jones. 6th ed. Singapore: Wiley-Blackwell; 2024. pp. 197–209.

[10] Sharman JE, Tan I, Stergiou GS. Automated ‘oscillometric’ blood pressure measuring devices: how they work and what they measure. J Hum Hypertens. 2023;37:93–100. 35637256 10.1038/s41371-022-00693-x PMC9957730

[11] Vachon C, Belanger MC, Burns PM. Evaluation of oscillometric and doppler ultrasonic devices for blood pressure measurements in anesthetized and conscious dogs. Res Vet Sci. 2014;97:111–7. 24924217 10.1016/j.rvsc.2014.05.003

[12] da Cunha AF, Saile K, Beaufrère H, Wolfson W, Seaton D, Acierno MJ. Measuring level of agreement between values obtained by directly measured blood pressure and ultrasonic doppler flow detector in cats. J Vet Emerg Crit Care. 2014;24:272–8. 10.1111/vec.12161 24697972

[13] Cerejo SA, Teixeira-Neto FJ, Garofalo NA, Pimenta EL, Zanuzzo FS, Klein AV. Effects of cuff size and position on the agreement between arterial blood pressure measured by doppler ultrasound and through a dorsal pedal artery catheter in anesthetized cats. Vet Anaesth Analg. 2020;47:191–9. 32007443 10.1016/j.vaa.2019.11.001

[14] Barter LS, Epstein SE. Comparison of Doppler, oscillometric, auricular and carotid arterial blood pressure measurements in isoflurane anesthetized new Zealand white rabbits. Vet Anaesth Analg. 2014;41:393–7. 24571422 10.1111/vaa.12131

[15] Szaluś-Jordanow O, Czopowicz M, Świerk A, Szpinda O, Garncarz M, Mickiewicz M, et al. Oscillometric and doppler arterial blood pressure measurement in conscious goats. Can J Vet Res. 2018;82:244–8. 30363283 PMC6168019

[16] Harvey L, Knowles T, Murison PJ. Comparison of direct and doppler arterial blood pressure measurements in rabbits during isoflurane anaesthesia. Vet Anaesth Analg. 2012;39:174–84. 22356416 10.1111/j.1467-2995.2011.00685.x

[17] Kennedy MJ, Michele B. Agreement between doppler and invasive blood pressure monitoring in anesthetized dogs weighing < 5 kg. Am Anim Hosp Assoc. 2015;51:300–5. 10.5326/JAAHA-MS-6163 26355579

[18] Lapid R, Shilo-Benjamini Y. Immobilization of captive Nubian Ibex (*Capra nubiana*) with butorphanol-midazolam-medetomidine or butorphanol-azaperone-medetomidine and Atipamezole reversal. J Wildl Dis. 2009;45:468–80. 26056882 10.1638/2014-0202R1.1

[19] Brown SA, Atkins C, Bagley R, Carr A, Cowgill L, Davidson M, et al. Guidelines for the identification, evaluation and management of systemic hypertension in dogs and cats. J Vet Intern Med. 2007;21:542–58. 17552466 10.1892/0891-6640(2007)21[542:gftiea]2.0.co;2

[20] Trim CM, Hofmeister EH, Peroni JF, Thoresen M, et al. Evaluation of an oscillometric blood pressure monitor for use in anesthetized sheep. Vet Anaesth Analg. 2013;40:31–9. 10.1111/vaa.12018 23438032

[21] Almeida D, Barletta M, Mathews L, Graham L, Quandt J. Comparison between invasive blood pressure and a non-invasive blood pressure monitor in anesthetized sheep. Res Vet Sci. 2014;97:582–86. 25458506 10.1016/j.rvsc.2014.10.004

[22] Mosing M, Franz S, Iff I, Schwendenwein I, et al. Invasive Arterielle Blutdruckmessung mittels eines aneroiden systems beim rind. Schweiz Arch Tierheilkd. 2009;151:275–80. 19496047 10.1024/0036-7281.151.6.275

[23] Riebold TW, Evans AT. Blood pressure measurements in the anesthetized horse: comparison of four methods. Vet Surg. 1985;14:332–7.

[24] Adam M, Raekallio MR, Salla KM, Honkavaara JM, Männikkö S, Scheinin M, et al. Effects of the peripherally acting α2-adrenoceptor antagonist MK-467 on cardiopulmonary function in sheep sedated by intramuscular administration of Medetomidine and ketamine and reversed by intramuscular administration of Atipamezole. Am J Vet Res. 2018;79:921–32. 30153057 10.2460/ajvr.79.9.921

[25] Bland JM, Altman DG. Agreement between methods of measurement with multiple observations per individual. J Biopharm Stat. 2007;17:571–82. 17613642 10.1080/10543400701329422

[26] Carkeet A. A review of the use of confidence intervals for Bland-Altman limits of agreement in optometry and vision science. Optom Vis Sci. 2020;97:3–8. 31895271 10.1097/OPX.0000000000001465

[27] Gent TC, Schwarz A, Hatz LA, Gozalo-Marcilla M, Schauvliege S, Gasthuys F, et al. Evaluation of accuracy of invasive and non-invasive blood pressure monitoring in relation to carotid artery pressure in anaesthetized ponies. Pferdeheilkunde. 2015;31:33–8.

[28] Wilson KAT, Raisis AL, Drynan EA, Mosing M, Lester GD, Hayman J, et al. Agreement between invasive blood pressure measured centrally and peripherally in anesthetized horses. Vet Anaesth Analg. 2018;45:467–76. 29880276 10.1016/j.vaa.2018.02.006

[29] Hall JE, Hall ME. Overview of the circulation: pressure, flow, and resistance. In: Hall JE, Hall ME, editors. Guyton and hall textbook of medical physiology. Philadelphia: Elsevier; 2021. pp. 171–81.

[30] Haberman CE, Kang CW, Morgan JD, Brown SA. Evaluation of oscillometric and doppler ultrasonic methods of indirect blood pressure Estimation in conscious dogs. Can J Vet Res. 2006;70:211–7. 16850944 PMC1477936

[31] Bosiack AP, Mann FA, Dodam JR, Wagner-Mann CC, Branson KR. Comparison of ultrasonic doppler flow monitor, oscillometric, and direct arterial blood pressure measurements in ill dogs. J Vet Emerg Crit Care. 2010;20:207–15. 10.1111/j.1476-4431.2010.00520.x 20487248

[32] Aarnes TK, Hubbell JAE, Lerche P, Bednarski RM. Comparison of invasive and oscillometric blood pressure measurement techniques in anesthetized sheep, goats, and cattle. Vet Anaesth Analg. 2014;41:174–85. 24224756 10.1111/vaa.12101

[33] Izer J, Wilson R. Comparison of invasive and non-invasive blood pressure measurements in anesthetized female Dorset cross-bred lambs (*Ovis aries*). Res Vet Sci. 2020;132:257–61. 32688102 10.1016/j.rvsc.2020.07.004

[34] Giavarina D. Understanding Bland Altman analysis. Biochem Med. 2015;25:141–51. 10.11613/BM.2015.015 PMC4470095 26110027

